# Experiences in close relationships, interpersonal trust and OXTR expression in individuals with childhood maltreatment

**DOI:** 10.1186/s40479-026-00340-8

**Published:** 2026-03-23

**Authors:** Inga Niedtfeld, Marie Kolar née Hofmann, Johanna Hepp, Sara E. Schmitz, Sylvia Steinmann, Stephanie H. Witt

**Affiliations:** 1 Department of Psychosomatic Medicine and Psychotherapy, Central Institute of Mental Health, Medical Faculty Mannheim, Heidelberg University, Mannheim, Germany; 2 Mental mHealth Lab, Institute of Sports and Sports Science, Karlsruhe Institute of Technology, Karlsruhe, Germany; 3 Department of Genetic Epidemiology in Psychiatry, Central Institute of Mental Health, Medical Faculty Mannheim, University of Heidelberg, Mannheim, Germany; 4 Biobank of the Center for Innovative Psychiatric and Psychotherapeutic Research, Central Institute of Mental Health, Medical Faculty Mannheim, University of Heidelberg, Mannheim, Germany; 5 Department for Psychosomatics and Psychotherapeutic Medicine, Central Institute of Mental Health, PO-Box 12 21 20, 68072 Mannheim, Germany

**Keywords:** Childhood maltreatment, Oxytocin receptor (OXTR), Interpersonal trust, Distrust, Attachment anxiety, Attachment avoidance, Loneliness, Social cognition

## Abstract

**Background:**

Social cognition is essential for forming and maintaining intimate relationships. Individuals with a history of childhood maltreatment (CM) often show impairments in interpersonal functioning, including trust and romantic attachment. The oxytocin system has been implicated in social behavior, yet the role of oxytocin receptor (*OXTR*) gene expression in CM-related social functioning remains insufficiently understood. This study examined the impact of CM on social cognition in a large web-based sample and explored the association between CM and *OXTR* gene expression in a well-characterized clinical cohort.

**Methods:**

Both studies were preregistered (10.17605/OSF.IO/KNC2X), and data and code are available at OSF (10.17605/OSF.IO/4DZWK). Study 1 assessed the relationship between CM severity, interpersonal trust, and romantic attachment in a web-based sample (*N* = 252). Participants completed the Childhood Trauma Questionnaire, the Generalized Trust Scale, a behavioral distrust game, and the Experiences in Close Relationships Questionnaire. Study 2 analyzed *OXTR* gene expression in peripheral blood samples from *N* = 92 CM-exposed individuals and examined associations with self-reported CM severity. Exploratory analyses additionally included measures of interpersonal sensitivity, social functioning, and maladaptive personality traits.

**Results:**

In Study 1, greater CM severity was significantly associated with lower interpersonal trust (both self-reported and behavioral), and with higher levels of attachment-related anxiety and avoidance. In Study 2, no significant association was found between CM severity and *OXTR* gene expression in blood. However, exploratory analyses revealed that CM severity was associated with social interaction difficulties, while *OXTR* gene expression was associated with the maladaptive personality trait of detachment.

**Conclusions:**

The findings highlight robust associations between CM and impairments in trust as well as insecure romantic attachment. Although no direct link was found between CM and *OXTR* expression, future research should investigate gene–environment interactions using larger samples and alternative tissue sources. Interventions targeting trust and attachment processes may offer promising avenues to improve relational functioning in individuals with a history of CM.

**Supplementary Information:**

The online version contains supplementary material available at 10.1186/s40479-026-00340-8.

## Theoretical background

Childhood maltreatment (CM) constitutes a significant public health concern with profound and lasting effects on individuals’ psychological, social, and biological functioning. CM encompasses physical, emotional, and sexual abuse, as well as emotional and physical neglect [1]. Research has consistently demonstrated that early-life adversity is associated with an increased risk for various mental health disorders, including major depression [2–4], substance abuse [5, 6], personality disorders such as borderline personality disorder [7, 8] and psychopathy [9], antisocial behavior [10], self-harm and suicidality [11, 12], anxiety disorders [13], post-traumatic stress disorder [14], and comorbid presentations of depression and PTSD [15].

Beyond these well-documented mental health outcomes, CM also profoundly influences interpersonal functioning. Affected individuals often experience heightened fear of intimacy, emotional avoidance, and difficulties in forming trusting relationships [16–20]. Deficits in interpersonal trust and attachment insecurity are frequently observed in CM-exposed populations [21, 22]. These impairments may contribute to maladaptive relationship patterns, increased loneliness, and difficulties in forming and maintaining close interpersonal bonds, further exacerbating the risk for mental disorders [23, 24].

Interpersonal trust is a fundamental component of social relationships, influencing cooperation, reciprocity, and the ability to form secure attachment [25]. Trust deficits have been consistently observed in CM-exposed individuals, who display increased distrust, heightened threat sensitivity, and difficulties perceiving others as reliable [26–28]. Experimental studies using economic trust games have further demonstrated that CM-exposed individuals tend to exhibit reduced trusting behaviors and a greater expectation of betrayal compared to non-exposed individuals [29, 30].

Research suggests that CM can lead to avoidance of emotional closeness, difficulties expressing vulnerability, and fear of dependence on others [31]. In the context of romantic relationships, CM has been linked to attachment-related anxiety and avoidance, which are key indicators of insecure attachment styles [32]. Attachment-related anxiety involves heightened fear of abandonment and excessive partner dependence, while attachment-related avoidance involves discomfort with intimacy and preference for emotional independence [33]. Importantly, different aspects of insecure attachment were integrated within the Alternative DSM-5 Model for Personality Disorders. First, *intimacy* is a central aspect of the Level of Personality Functioning and describes the capacity for closeness, mutual connectedness, and enduring emotional bonds [31]. Second, the maladaptive personality trait *detachment* likewise reflects the tendency to maintain emotional and interpersonal distance and avoid close relationships [34].

With regards to relationship quality, a history of CM has been linked to increased relationship instability, reduced relationship satisfaction, and an increased risk for intimate partner violence [35, 36]. Moreover, accumulating evidence suggests that CM is associated with smaller social networks and increased loneliness [23, 24]. In turn, loneliness was negatively associated with perceived social support and predicted poorer mental health outcomes [37, 38]. Therefore, Barnes et al. [39] even proclaimed the idea of CM-exposed individuals being at risk of a self-perpetuating “circle of loneliness”. However, supportive romantic partners may buffer against early adversity’s negative consequences [40, 41].

Building on this interpersonal literature, we conceptualize trust as a contextual resilience/vulnerability factor in close relationships. Specifically, higher trust supports benign attributions, proximity seeking, and emotion regulation [25], whereas CM-related reductions in trust, as documented in behavioral paradigms [29, 30], may amplify hypervigilance and withdrawal. Accordingly, we expected moderation such that greater self-reported trust (and lower behavioral distrust) would weaken the positive association between CM and attachment anxiety/avoidance.

A growing body of research suggests that disruptions in the oxytocin system may underlie some of these social impairments. Oxytocin (OXT), a neuropeptide produced in the hypothalamus, is recognized for its role in social bonding, trust, intimacy, and emotional regulation [42, 43]. The oxytocin receptor (*OXTR*) has been implicated in empathy, attachment, and social sensitivity [44, 45]. Studies using intranasal OXT application have reported increased prosocial behavior, including trust [46, 47], empathy [48], altruism [49] – as well as positive effects on couple communication [50].

Extending this rationale to a molecular level, individual differences in oxytocin-system signalling may shape affiliative processing and social sensitivity [44, 45]. Consistent with experimental evidence that intranasal OXT can enhance trust and modulate socio-affective responses [46, 47, 50], we therefore predicted that higher OXTR expression would attenuate the negative association between CM and trust. It could also be discussed that a mediation pathway (CM leading to lower OXTR leading to lower trust) is theoretically plausible; however, it cannot be identified robustly in cross-sectional data and was therefore not preregistered.

Epigenetic mechanisms, especially OXTR DNA methylation, may help explain how CM shapes oxytocin-system functioning. Several studies suggest that CM is associated with higher OXTR methylation [51, 52], which has been linked to social and emotional dysregulation [53, 54] and to impairments in social bonding and intimacy [55, 56]. In a meta-analysis comprising 15 independent samples early adversity was found to be associated with higher OXTR DNA methylation levels [57]. However, studies investigating the relationship between CM and the oxytocin system have yielded mixed findings, i.e. either the associations were non-significant after correction for multiple testing [58] or no association was found [59]. While methylation is one layer of regulation, it does not deterministically translate into mRNA levels. Evidence suggests that methylation–expression coupling is region- and locus-specific and can be modest in peripheral blood [60, 61]. In this study, we quantified OXTR gene expression in peripheral blood cells as a more proximal molecular readout to examine its association with dimensional CM severity. To our knowledge, human studies linking CM to OXTR gene expression in peripheral blood are limited [62].

The present study aims to address two research gaps, testing (i) whether CM relates to lower interpersonal trust and more insecure romantic relationship attachment and (ii) whether CM severity is associated with alterations in *OXTR* gene expression. Given the established role of oxytocin in social behavior, elucidating the potential link between CM and *OXTR* expression may provide valuable insights into the biological mechanisms underlying CM-related social impairments.

### The current studies

The present research was conducted within the framework of the DFG-funded research training group *Impact of Adverse Childhood Experiences on Psychosocial and Somatic Conditions across the Lifespan (RTG2350)*, which investigates the neurobiological, somatic, and psychosocial consequences of CM. Both studies were preregistered on OSF [63].

We conducted two complementary studies. Study 1 aimed to replicate findings on the effects of CM on interpersonal trust, examined effects on romantic relationship attachment, and explored the moderating role of trust. Interpersonal trust was assessed using both self-report measures and a behavioral distrust paradigm (distrust game). Romantic relationship attachment was assessed through levels of anxiety and avoidance. Study 2 investigated associations between CM severity and *OXTR* gene expression levels in peripheral blood samples to explore potential biological correlates of CM-related social impairments. We initially planned to assess all constructs within the same sample; however, due to recruitment difficulties and restrictions during the COVID-19 pandemic, we were unable to obtain blood samples from participants in Study 1, necessitating a separate sample.

We hypothesized that greater CM severity would be associated with (H1) lower interpersonal trust and (H2) higher levels of anxious and avoidant romantic relationship attachment. Furthermore, we hypothesized (H3) that interpersonal trust would moderate the relationship between CM and romantic relationship attachment. Finally, we hypothesized that severity of childhood trauma is associated with the level of *OXTR* expression in peripheral blood cells and might moderate trust (H4). Please note that we preregistered moderation analyses to probe whether higher trust or lower behavioral distrust attenuate CM-related attachment insecurity, and whether OXTR expression modifies CM–trust links. This operationalizes trust and OXTR as contextual modifiers (buffering/vulnerability factors) rather than transmission mechanisms (mediators), which would have required a longitudinal design. The preregistered hypotheses were as follows:

- H1a: The severity of childhood trauma (CTQ sum score) is negatively associated with the level of self-reported interpersonal trust (GTS).
- H1b: The severity of childhood trauma (CTQ sum score) is positively associated with the level of behavioral distrust (distrust game).
- H2a: The severity of childhood trauma (CTQ sum score) is positively associated with the levels of self-reported anxiety in intimate relationships (ECR-R scale anxiety mean score).
- H2b: The severity of childhood trauma (CTQ sum score) is positively associated with the levels of self-reported avoidance in intimate relationships (ECR-R scale avoidance mean score).
- H3a: Self-reported interpersonal trust (GTS) or behavioral distrust (distrust game) moderate the effect of ACE (CTQ sum score) on anxiety (ECR-R scale anxiety mean score) in intimate relationships.
- H3b: Self-reported interpersonal trust (GTS) or behavioral distrust (distrust game) moderate the effect of ACE (CTQ sum score) on avoidance (ECR-R scale avoidance mean score) in intimate relationships.

Study 2 focused on genetic correlates of CM by quantifying the expression of the OXTR gene in individuals who experienced CM. The preregistered hypotheses read as follows:

- H4a: The severity of childhood trauma (CTQ sum score) is negatively associated with the level of OXTR expression in peripheral blood cells.
- H4b: The effect of Childhood Trauma (CTQ sum score) on self-reported interpersonal trust (GTS) / behavioral distrust (distrust game) is moderated by the level of OXTR expression in peripheral blood cells.

In addition to these preregistered hypotheses, we registered planned exploratory analyses concerning the effects of loneliness alongside trust and attachment, in order to contextualize interpersonal functioning.

## Methods study 1

The study hypotheses were preregistered on Nov 25, 2021, prior to data collection [63]. As outlined in the preregistration, we pooled data from two sources to ensure a representative sample with a broad range of CM severity: a social media sample (subsample 1) and a sample recruited within the research training group RTG2350 at the Central Institute of Mental Health (CIMH) in Mannheim, Germany (subsample 2). Ethics approval was granted by the Medical Ethics Committee II of the Medical Faculty Mannheim, Heidelberg University (protocol numbers 2021-512 and 2018-562N-MA). The study procedure was identical for both subsamples, with the only difference being that participants in subsample 2 received a personalized link for their data with existing self-report measures from the RTG database and for receiving financial compensation.

A priori power analysis using G*Power [64] for Hypothesis 1 indicated a required sample size of at least 274 participants to detect small effects (f^2^ = 0.08) with an alpha level of *α* = 0.01 and power of 0.99. Based on this, we set a target sample size of 280 and stopped recruitment upon reaching *N* = 285 participants (n₁ = 256, n₂ = 29). In accordance with our preregistered exclusion criteria, we excluded 25 participants who had answered fewer than four items on at least one of the five CM questionnaire subscales, which is necessary to compute a valid sum score. Additionally, eight participants were excluded due to zero variance in their responses on one of the questionnaires or the distrust game. After exclusions, the final sample comprised *N* = 252 participants.

For subsample 1, data were collected online between September 2021 and February 2023. The study link was distributed via CIMH social media channels and other platforms, groups, and forums related to mental health topics. In order to reach individuals with higher levels of childhood adversity, we specifically shared the study in online communities related to CM, borderline personality disorder (BPD), and post-traumatic stress disorder (PTSD), as individuals with BPD or PTSD exhibit higher rates of CM [65, 66]. The study was introduced as examining the impact of CM on trust and romantic relationships. Participation was open to anyone aged 18 or older and currently involved in a romantic relationship. As an incentive, participants could enter a lottery to win one of fifty 20-Euro gift cards redeemable at an online store (www.wunschgutschein.de).

A total of 258 participants were recruited online who completed all mandatory study items. After applying preregistered exclusion criteria, subsample 1 comprised 225 participants. Most participants (*n* = 171) were recruited via social media platforms such as Facebook and Instagram, including nine from trauma- and BPD-related Facebook groups. An additional 54 participants were recruited via the CIMH’s own social media channels.

Additional participants for subsample 2 were recruited via central recruitment within RTG2350 from August 2022 until February 2024. Recruitment was delayed due to pandemic-related circumstances. After an initial screening to confirm eligibility for the RTG (e.g., age ≥ 18, experience of CM, no current substance abuse), participants underwent clinical assessment for CM and other diagnoses using the German *Structured Clinical Interview for DSM-5* [67], conducted by trained diagnosticians. Those who reported being in a current romantic relationship were invited to complete the online study via an individual link. Participants in subsample 2 received compensation in line with RTG guidelines (€12/hour). A total of 29 individuals completed all required items, with 27 remaining in the final sample after exclusions.

### Procedure

This web-based study was conducted using the open-source survey tool SoSciSurvey (https://www.soscisurvey.de), which is available free for non-commercial research and complies with German data privacy regulations. The questionnaire archive is available at OSF [68]. Upon accessing the study link, participants received detailed information about the study’s content and duration, including a disclaimer regarding potentially distressing content. They were informed that all data would be stored and processed anonymously. Contact information for the first and last author was provided, and participants had the option to download the study information.

Participants then completed self-report questionnaires on interpersonal trust, romantic relationship attachment (anxiety and avoidance), and social isolation, followed by the distrust game and a questionnaire on CM. A list of all questionnaire items (German versions) is provided in the supplementary materials. To minimize missing data, a forced-response format was used for all items except the CM questionnaire and demographics, where responses were optional for ethical reasons. At the end of the study, participants could voluntarily provide their email address and consent to be contacted regarding the lottery (subsample 1 only), the study results, or both. Email addresses were stored separately from study data using a built-in SoSciSurvey feature to ensure anonymity.

### Self-report questionnaires

*Childhood Maltreatment.* The type and severity of CM were assessed with the Childhood Trauma Questionnaire (CTQ; Bernstein et al., 1994; German version: Bader et al., 2009). The CTQ is a self-report instrument that retrospectively assesses experiences of maltreatment before age 18. It includes five subscales: Emotional Abuse (EA), Physical Abuse (PA), Sexual Abuse (SA), Emotional Neglect (EN), and Physical Neglect (PN), each consisting of five items in the 25-item short version used in this study. Participants rated how often they experienced each type of maltreatment (e.g., “When I was growing up, my family said hurtful things”) on a 5-point Likert scale (1 = not at all to 5 = very often). We calculated both subscale scores (range: 5–25) and a total score (range: 25–125), with higher scores indicating more severe CM. The CTQ shows good internal consistency (EN and SA: α = 0.90; EA: α = 0.82; PA: α = 0.83), except for the PN subscale (α = 0.49), and high test-retest reliability, ranging from 0.74 to 0.94 [69]. The instrument correlates well with therapists’ clinical ratings (highest correlation for SA: *r* = .75) and has demonstrated measurement equivalence across clinical and non-clinical populations [70]. The German version was validated by Klinitzke et al. [71].

*Romantic Relationship Attachment.* Romantic attachment was measured using the *Experiences in Close Relationships–Revised* (ECR-R; 33), a 36-item self-report instrument comprising two subscales: Attachment-related anxiety and attachment-related avoidance. Each subscale contains 18 items (e.g., Anxiety: “I’m afraid that I will lose my partner’s love”; Avoidance: “I prefer not to show my partner how I feel deep down.“). Participants responded on a 7-point Likert scale (1 = strongly disagree to 7 = strongly agree). The ECR-R shows excellent psychometric properties, including high retest reliability (*r* = .93) and internal consistency (Anxiety: α = 0.93; Avoidance: α = 0.91) [72], confirmed in a German validation study [73].

*Loneliness.* Loneliness was assessed using the *University of California (Los Angeles) Loneliness Scale* [74], in the revised German version by Döring et al. [75]. This 20-item instrument captures subjective feelings of loneliness and social isolation. Items (e.g., “I have nobody to talk to”) are rated on a 5-point Likert scale (1 = I never feel this way to 5 = I always feel this way), with higher scores indicating greater loneliness. The third version of the UCLA scale demonstrates high internal consistency (α = 0.89–0.94) and one-year test–retest reliability (*r* = .73) [76].

*Interpersonal Trust.* Interpersonal trust was assessed using the *Generalized Trust Scale* [77], translated into German by Igarashi et al. [78]. Participants rated their agreement with six statements (e.g., “Most people are trustworthy”) on a 5-point Likert scale (1 = strongly disagree to 5 = strongly agree). A mean score was calculated, with higher values indicating greater trust.

*Behavioral Distrust*. To assess trust behaviorally, participants completed a hypothetical economic exchange task known as the distrust game (DG), originally developed by McEvily et al. [79] and refined by Thielmann et al. [80]. We used the version applied by Hepp et al. [29], which includes standardized facial stimuli [81]. Participants estimated how much money a virtual interaction partner would take from an initial 50 Euros, selecting any whole number between 0 and 50. Higher amounts indicated greater distrust. A total of 21 faces were presented, each representing one of seven trustworthiness levels (− 3 to + 3), with each level presented three times.

### Data analysis

The dataset of study 1 and the analysis code are available at OSF [68]. For the preregistered analyses, we used the CTQ total score to quantify CM severity. Mean scores were calculated for the GTS, the two ECR-R subscales, and the UCLA scale, representing interpersonal trust, attachment-related anxiety and avoidance, and loneliness, respectively. Behavioral distrust was calculated by averaging participants’ estimates across the three presentations of each of the seven trustworthiness levels in the DG.

To test preregistered hypotheses and conduct exploratory analyses involving the UCLA, we ran linear regression models. To enhance the interpretability of regression coefficients, all continuous predictors were grand-mean centered. All models included the following preregistered covariates: age (grand mean centered), gender (1 = female, 0 = other), sexual orientation (1 = heterosexual, 0 = other), education level (1 = university entrance degree, 0 = other), and migration background (1 = with migration background up to second generation, 0 = without). For hypotheses H2 and H3, which focused on romantic attachment as the dependent variable, we additionally included relationship length (grand mean centered) as a covariate. Beyond these preregistered covariates, we did not add clinical diagnoses or psychopathology as covariates since this would not align with the dimensional design of our study, and would raise concerns about multicollinearity (since CTQ and psychopathology are known to be correlated). As preregistered, we applied a uniform two‑tailed α = 0.01 to all confirmatory tests. This a-priori choice reflects that each construct was tested via two closely related operationalizations (e.g., self-reported trust and behavioral distrust; attachment anxiety and attachment avoidance), which are non‑independent.

### Use of generative AI

We used ChatGPT (OpenAI) to assist with copy editing and minor R code refactoring for figure generation. The tool did not contribute to study design, data collection, data analysis, or interpretation of the results. All outputs were verified by the authors, who take full responsibility for the content.

## Results study 1

### Sample demographics

The final sample included participants aged 18 to 65 years, with most being young adults (M = 31.2, SD = 9.2). The majority identified as women (92.5%), heterosexual (75.4%), had at least a high school diploma (57.9%), and reported no history of migration (78.6%). Participants reported an average relationship length of 6.9 years (SD = 7.7, range = 0–41) and relatively high relationship satisfaction (M = 7.4, SD = 2.0). Detailed sociodemographic information is presented in Supplemental Table S1.

The average CTQ total score was M = 55.3 (SD = 21), with a median of 52.5, indicating a right-skewed distribution (range: 25–108). Subsample 2 reported higher CTQ scores than subsample 1. According to the severity classification by Häuser et al. [82], more than half of the sample reported moderate to severe levels of emotional abuse (61.9%), followed by emotional neglect (54.0%), physical neglect (39.0%), sexual abuse (34.1%), and physical abuse (23.0%). One in five participants (23.0%) reported severe scores on more than two subscales.

The mean score on the GTS was M = 3.0 (SD = 0.8), indicating a neutral level of self-reported trust. In the DG, participants estimated that opponents would take an average of 23 Euros (SD = 11.0) from their 50-Euro endowment. The ECR-R anxiety mean was M = 4.0 (SD = 1.2); avoidance was M = 3.0 (SD = 1.3). Distributions for both subscales were right-skewed. The UCLA loneliness score averaged M = 2.4 (SD = 0.9), reflecting a moderate level of perceived loneliness.

Pearson correlations between all key variables and relationship satisfaction are displayed in Supplemental Table S2. All correlations were in the expected direction. Higher CTQ scores were associated with lower trust (both GTS and DG), and higher levels of attachment-related anxiety and avoidance (ECR-R). UCLA loneliness scores were significantly correlated with all other variables. Effect sizes ranged from small to moderate. Cronbach’s α for the measures ranged from 0.87 (GTS) to 0.95 (ECR-R Avoidance), indicating high internal consistency.

### Hypothesis 1: Childhood Maltreatment and Interpersonal Trust

To test Hypothesis 1, we conducted multiple regression analyses predicting self-reported interpersonal trust (GTS; Model 1) and behavioral distrust (DG; Model 2) based on the CTQ total score, while controlling for preregistered covariates.

**Fig. 1:**
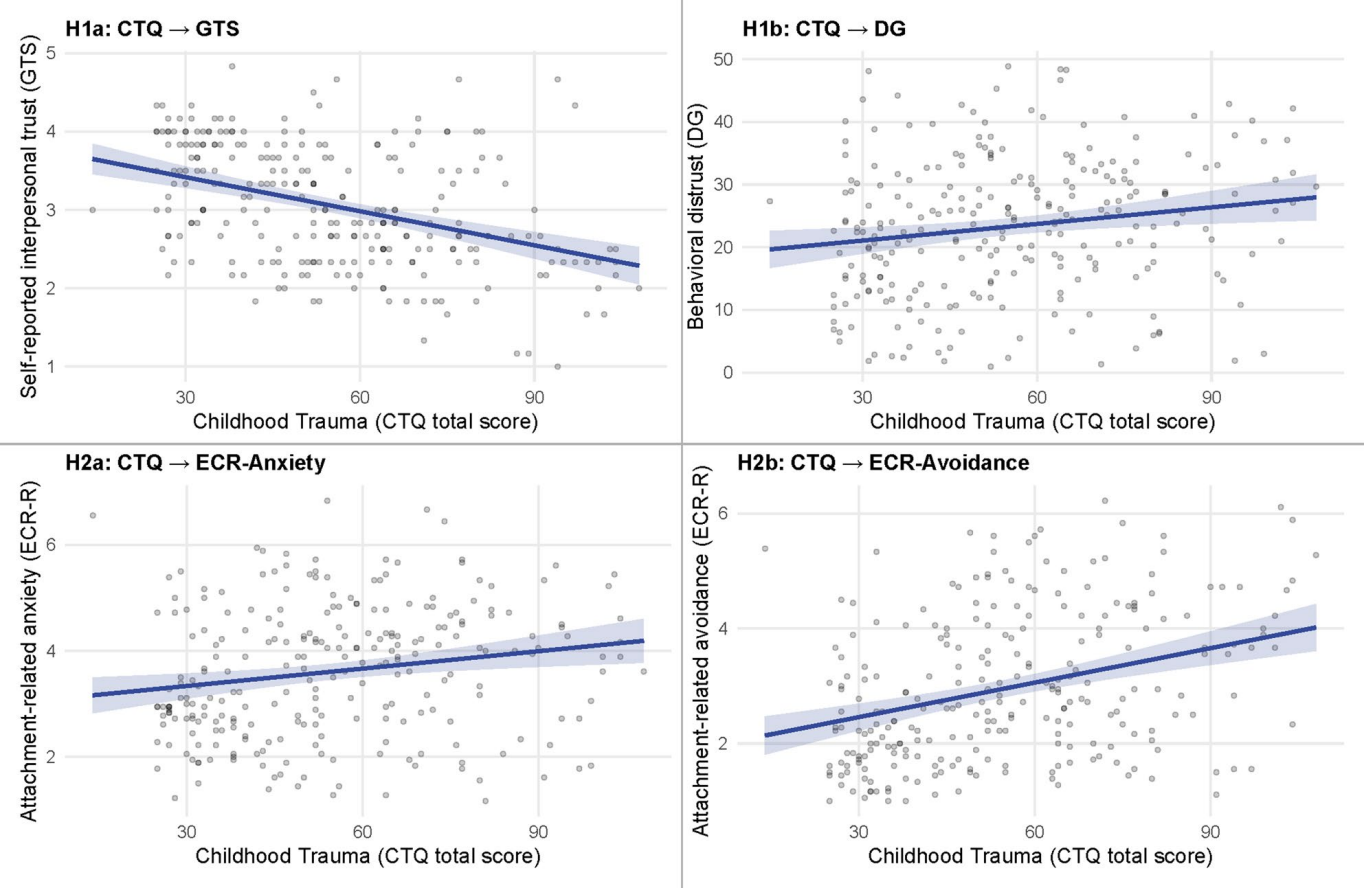
Associations between childhood maltreatment and trust (Hypothesis 1), and between childhood maltreatment and romantic relationship attachment (Hypothesis 2). *Note.* CTQ = Childhood Trauma Questionnaire, GTS = General Trust Scale, DG = Distrust Game, ECR = Experiences in Close Relationships. Lines show model predictions with 95% CIs; points are raw observations

In model 1, higher CTQ scores were significantly associated with lower GTS scores (b = -0.01, *p* < .01), indicating that greater CM severity predicted lower self-reported interpersonal trust. The model explained 21% of the variance (R² = 0.21). In model 2, a higher CTQ total score significantly predicted greater DG scores (b = 0.09, *p* < .01), indicating that higher CM severity was associated with greater behavioral distrust. This model accounted for 8% of the variance (R² = 0.08). No other covariates were significant in either model. Model estimates are reported in Table 1, for visualization see Fig. 1.

**Table 1:** Linear multiple regression models for the test of Hypothesis 1

Predictors	Model 1: GTS		Model 2: DG	
	b [95% CI]	β [95% CI]	b [95% CI]	β [95% CI]
Intercept	3.10 [2.74, 3.46]		18.08 [12.63, 23.53]	
CTQ (total)	**-0.01 [-0.02**,** -0.01]**	**-0.40 [-0.51**,** -0.28]**	**0.09 [0.02**,** 0.15]**	**0.17 [0.05**,** 0.29]**
Age	-0.01 [-0.02, 0.00]	-0.11 [-0.23, 0.02]	0.15 [-0.01, 0.31]	0.13 [-0.01, 0.26]
Gender female	-0.18 [-0.50, 0.14]	-0.06 [-0.17, 0.05]	5.97 [1.06, 10.88]	0.15 [0.03, 0.27]
Sexual orientation	0.21 [0.00, 0.42]	0.12 [0.00, 0.23]	0.07 [-3.10, 3.24]	0.00 [-0.12, 0.13]
Educational level	-0.05 [-0.24, 0.13]	-0.03 [-0.15, 0.08]	0.28 [-2.60, 3.17]	0.01 [-0.11, 0.14]
Migration history	-0.12 [-0.33, 0.10]	-0.06 [-0.18, 0.05]	-2.08 [-5.40, 1.24]	-0.08 [-0.20, 0.05]

Note. CTQ = Childhood Trauma Questionnaire, GTS = General Trust Scale, DG = Distrust Game. The covariates were dummy-coded: gender (1 = female, 0 = other), sexual orientation (0 = heterosexual), educational level (1 = university entrance degree, 0 = other). Significant results *p* < .01 in bold

### Hypothesis 2: Childhood Maltreatment and Romantic Relationship Attachment

#### Hypothesis 2

was tested in two separate regression models, with attachment-related anxiety (Model 3) and avoidance (Model 4) as the respective outcome variables.

In model 3, both a higher CTQ total score (b = 0.01, *p* < .01) and lower relationship length (b = -4.13, *p* < .01) were significant predictors of greater attachment-related anxiety. The model explained 9% of the variance (R² = 0.09).

In model 4, a higher CTQ total score was also a significant predictor of more attachment-related avoidance (b = 0.02, *p* < .01), with the model explaining 17% of the variance (R² = 0.17). No other covariates significantly contributed to either model. Full estimates are shown in Table 2, for visualization see Fig. 1.

**Table 2:** Linear multiple regression models for the test of Hypothesis 2

Predictors	Model 3: ECR-Anxiety		Model 4: ECR-Avoidance	
	b [95% CI]	β [95% CI]	b [95% CI]	β [95% CI]
Intercept	**3.17 [2.55**,** 3.79]**		**3.07 [2.46**,** 3.68]**	
CTQ (total)	**0.01 [0.00**,** 0.02]**	**0.19 [0.06**,** 0.31]**	**0.02 [0.01**,** 0.03]**	**0.33 [0.21**,** 0.45]**
Age	0.02 [-0.01, 0.04]	0.14 [-0.04, 0.32]	**0.03 [0.00**,** 0.05]**	**0.19 [0.02**,** 0.36]**
Gender	**0.59 [0.03**,** 1.15]**	**0.13 [0.01**,** 0.25]**	0.03 [-0.52, 0.58]	0.01 [-0.11, 0.12]
Sexual orientation	-0.18 [-0.54, 0.18]	-0.06 [-0.19, 0.06]	-0.18 [-0.54, 0.17]	-0.06 [-0.18, 0.06]
Educational level	0.02 [-0.31, 0.35]	0.01 [-0.12, 0.13]	0.05 [-0.27, 0.38]	0.02 [-0.10, 0.14]
Migration history	0.15 [-0.23, 0.53]	0.05 [-0.07, 0.17]	-0.06 [-0.43, 0.31]	-0.02 [-0.14, 0.10]
Relationship length	**-4.13 [-6.95**,** -1.31]**	**-0.25 [-0.43**,** -0.08]**	0.40 [-2.37, 3.18]	0.02 [-0.14, 0.19]

Note. CTQ = Childhood Trauma Questionnaire, GTS = General Trust Scale, DG = Distrust Game, ECR = Experiences in Close Relationships. The covariates were dummy-coded: gender (1 = female, 0 = other), sexual orientation (0 = heterosexual), educational level (1 = university entrance degree, 0 = other). Significant results *p* < .01 in bold

### Hypothesis 3: Moderation by trust and distrust

To test Hypothesis 3, we explored whether self-reported interpersonal trust (GTS; Models 5 and 6, see Supplemental Table S3) or behavioral distrust (DG; Models 7 and 8, see Table S4) moderated the association between CM severity and romantic relationship attachment. No significant interaction effects were found in any of the models, suggesting that we found no evidence for a moderation effect of self-reported trust or behavioral distrust on the relationship between CM and attachment-related anxiety or avoidance. Thus, Hypothesis 3 was not supported, see Supplemental Tables S3 and S4.

### Exploratory analyses: Predictors of loneliness

We explored the role of CM, interpersonal trust, romantic attachment, and relationship satisfaction in predicting loneliness, as measured by the UCLA mean score. In a multiple regression model including all theoretically relevant predictors, a substantial proportion of the variance in loneliness was explained (R² = 0.62). Significant independent predictors of higher loneliness were higher attachment-related anxiety (b = 0.17, *p* < .01), higher attachment-related avoidance (b = 0.22, *p* < .01), and lower self-reported trust (b = –0.34, *p* < .01). In contrast, CM severity and relationship satisfaction did not contribute uniquely to the prediction of loneliness when interpersonal variables were accounted for. For details on model estimates, see Supplemental Table S5.

## Methods study 2

This study was preregistered as an update to Study 1 on July 22, 2022, on OSF [63], prior to processing and analyzing the blood samples. As in Subsample 2 of Study 1, participants were recruited within the Research Training Group “Impact of Adverse Childhood Experiences on Psychosocial and Somatic Conditions Across the Lifespan” (RTG2350). Ethics approval was granted by the Medical Ethics Committee II of the Medical Faculty Mannheim, Heidelberg University (protocol no. 2021 − 512 and 2018–562 N-MA).

As preregistered, we recruited 94 participants who provided informed consent for blood sampling (1× EDTA, 2× PAXgene^®^ tubes). Participants also completed the standard RTG2350 survey battery, including demographic information and self-report questionnaires such as the Childhood Trauma Questionnaire (CTQ). Two participants were excluded due to incomplete responses on the CTQ (fewer than four items on any subscale), resulting in a final sample of 92 individuals. The procedures for blood preparation and gene expression analysis are detailed below, based on protocols similar to that used by Cosentino et al. [83]. Subsequently, we describe the self-report instruments.

### OXTR expression analysis

Blood samples were collected in RNA-stabilizing PAXgene^®^ tubes (BD Biosciences) and stored in the Biobank of the CIPP core facility of the Central Institute of Mental Health (CIMH) at − 80 °C until processing. RNA was isolated using the PAXgene^®^ Blood RNA Kit (Qiagen) following standard protocols. RNA quantity was assessed by optical density using a NanoDrop™ Spectrophotometer (Thermo Fisher Scientific). To assess RNA degradation, microfluidic RNA 6000 Pico chips (Agilent Technologies) were used with the Agilent 2100 Bioanalyzer System, which combines capillary electrophoresis with laser-induced fluorescence detection. Inclusion criteria for sample quality were as follows: an optical density ratio of 1.9–2.2 (260 nm/280 nm), RNA integrity number (RIN) > 8.0, and no detectable genomic DNA contamination on electropherograms. All samples met these criteria and were retained for analysis.

For quantification of oxytocin receptor (OXTR) gene expression, RNA was reverse-transcribed to complementary DNA (cDNA) using the high capacity cDNA reverse transcription kit with random primers (Applied Biosystems, Thermo Fisher Scientific) following the manufacturer’s standard protocol. Quantitative polymerase chain reaction (qPCR) was performed using the TaqMan Fast Advanced Master Mix (Applied Biosystems) with the QuantStudio^(TM)^ 7 Flex System following the manufacturer’s standard protocol. *OXTR* gene expression was measured using the TaqMan OXTR-Assay (Hs00168573_m1), Actin Beta (ACTB) served as internal reference, measured with the TaqMan Gene Expression Assay (Hs01060665_g1). All analyses were carried out in triplicate. Results were processed using QuantStudio Software v1.7.2, calculating quantification cycles (Cq) for both OXTR and ACTB. Cq values are inversely related to nucleic acid concentration (lower Cq indicates higher expression). Normalized OXTR expression was calculated using delta Cq (average Cq OXTR minus Cq ACTB).

### Self-report questionnaires

*Childhood Maltreatment.* The Childhood Trauma Questionnaire (CTQ; Bernstein et al., 1994; German version: Bader et al., 2009) was used as described in the methods section of Study 1.

*Psychopathology.* The Brief Symptom Inventory [84, 85] consists of 53 items capturing psychological distress across nine domains. We focused on the Interpersonal Sensitivity (INS) subscale, comprising four items assessing distress related to feelings of inferiority, social discomfort, and perceived hostility. Participants rated distress over the past 7 days on a 5-point scale (1 = not at all to 5 = extremely). The German version of the BSI has demonstrated good internal consistency (Cronbach’s *a* between 0.63 and 0.93) and convergent validity Spitzer et al. [86]. *Mental Health.* The 36-item version of the World Health Organization Disability Assessment [87] assesses disability across six domains. We focused on Domain 4 (social interaction), which includes five items addressing difficulties in social engagement over the past 30 days. Items are rated on a 5-point scale (1 = none to 5 = severe). Normative evaluations are available for interpretation [88].

*Maladaptive Personality Traits*. We used the short form of the Personality Inventory for DSM-5 [89–91], consisting of 100 items that assess 25 maladaptive personality facets. Responses were given on a 4-point Likert scale (0 = very false to 3 = very true). We focused on the Detachment domain, reflecting social withdrawal and anhedonia. The short form demonstrates high internal consistency (Cronbach’s α ≈ 0.85), and normative data for interpretation are available [92].

### Data analysis

The dataset of study 2 and the analysis code are available at OSF[68]. We used delta Cq as the outcome measure for OXTR expression. Predictor variables included CTQ total scores (CM severity), BSI-INS (interpersonal sensitivity), WHODAS Domain 4 (social interaction difficulties), and PID-5 Detachment (social withdrawal).

To test preregistered Hypothesis 4a, that greater CM severity would be associated with altered OXTR expression, we performed linear regression analyses. All continuous predictors were grand-mean centered to enhance the interpretability of regression coefficients.

Due to limited survey responses, we were unable to test preregistered Hypothesis 4b (moderation by OXTR expression). Instead, we conducted exploratory correlation analyses examining associations between OXTR expression and psychological functioning (BSI, WHODAS, PID-5). These analyses were restricted to participants with complete data across relevant variables, resulting in a final analytic sample of *n* = 75. Again, we applied an alpha level of α = 0.01.

### Use of generative AI

We used ChatGPT5 (OpenAI) to assist with copy editing and minor R code refactoring for figure generation. The tool did not contribute to study design, data collection, data analysis, or interpretation of the results. All outputs were verified by the authors, who take full responsibility for the content.

## Results study 2

### Sample demographics

Participants in the final sample (*N* = 92) ranged in age from 18 to 60 years (M = 31.18, SD = 10.89). The majority identified as female (*n* = 62, 67.4%), and *n* = 22 (23.9%) as male. For detailed demographic information, see Supplemental Table S6. Current diagnoses were assessed using the german *Structured Clinical Interview for DSM-5* [67], conducted by trained diagnosticians; see Supplemental Table S7. The majority of participants met criteria for current mood disorders, with 62.0% diagnosed with major depressive disorder. Posttraumatic stress disorder (41.3%), social anxiety disorder (20.7%), and eating disorders (20.7%) were also prevalent. Personality disorders were not assessed in this study.

OXTR expression, measured via delta Cq scores, was normally distributed (M = 15.00, SD = 0.60);Shapiro-Wilk test: W = 0.994, *p* = .96; skew = − 0.22, kurtosis = 0.03). CTQ total scores indicated moderate exposure to CM (M = 57.64, SD = 20.81; range = 25–109). Emotional neglect (M = 15.40, SD = 6.05) and emotional abuse (M = 14.75, SD = 6.30) were the most frequently reported forms of maltreatment.

Psychopathology levels were elevated across multiple domains. The Global Severity Index of the BSI averaged M = 98.08 (SD = 35.10), with the Interpersonal Sensitivity subscale showing M = 8.10 (SD = 3.22). Functional impairment was highest in the WHODAS domains of participation (M = 19.67, SD = 8.49) and life activities (M = 16.93, SD = 6.90). Social interaction difficulties (WHODAS Domain 4: Getting Along) were moderate (M = 7.77, SD = 3.44), and the total disability score was M = 69.42 (SD = 25.50).

With regard to maladaptive personality traits (PID-5), the Detachment domain score (M = 0.94, SD = 0.60), Negative affectivity (M = 1.33, SD = 0.68) and disinhibition (M = 1.11, SD = 0.56) were elevated as compared to antagonism (M = 0.57, SD = 0.50). Descriptive statistics and intercorrelations for all instruments are provided in the Supplemental Material (see supplemental Table S8).

### Descriptive correlations

Correlation analysis yielded small but consistent associations between OXTR expression (delta Cq score) and PID-5 Detachment (*r* = –.22, *p* = .041), as well as with its facets: interpersonal avoidance (*r* = –.21, *p* = .054), withdrawal (*r* = –.24, *p* = .030), and restricted affectivity (*r* = –.26, *p* = .019). While these effects did not meet the pre-specified significance threshold (*p* < .01), their consistency suggests a potentially meaningful pattern that warrants further investigation.

Childhood trauma severity (CTQ total score) was significantly correlated with several psychological outcomes. Specifically, higher CTQ scores were positively associated with BSI Interpersonal Sensitivity, WHODAS social interaction difficulties, and PID-5 Detachment. These associations suggest that increased exposure to CM was related to greater difficulties in interpersonal relationships. For details, see Supplemental Table S8.

### Hypothesis 4: Childhood trauma and OXTR expression

To test Hypothesis 4a, we performed a linear regression with CTQ total score as the predictor and *OXTR* expression (delta Cq) as the outcome. Results indicated no significant association between childhood trauma severity and *OXTR* expression (R² = 0.008, F_(1, 90)_ = 0.74, *p* = .392). Due to limited survey responses, we were unable to test preregistered Hypothesis 4b (moderation by OXTR expression).

### Exploratory analysis: OXTR expression and PID-5 detachment

In an exploratory linear regression, we examined whether *OXTR* expression was associated with maladaptive detachment traits (PID-5 Detachment domain). Results revealed a negative association (R² = 0.0499, F_(1, 82)_ = 4.31, *p* = .04), indicating that lower *OXTR* expression was associated with higher levels of detachment. However, these effects did not reach our conservative threshold for statistical significance at *p* < .01.

## Discussion

This paper presents two studies that aimed to investigate correlates of CM, specifically its interpersonal dysfunction (Study 1) and genetic correlates (Study 2). Previous research has often focused on highly traumatized individuals diagnosed with post-traumatic stress disorder [93] or specific CM subtypes (e.g., sexual abuse) [94], while neglect and milder forms remained underrepresented despite high prevalence [95]. Additionally, the role of interpersonal trust in romantic relationships of CM-exposed individuals has been understudied. Study 1 (*N* = 252) addressed these gaps by examining CM effects on interpersonal trust and romantic attachment, testing trust as a potential moderator. In line with our hypotheses H1 and H2, higher CM levels were associated with lower self-reported trust, increased behavioral distrust, and higher attachment anxiety and avoidance. However, we found no evidence for trust’s moderating effect (H3). Exploratory analyses revealed loneliness associations with all key variables.

To explore potential biological mechanisms linking CM and its adverse outcomes, Study 2 (*N* = 92) focused on OXTR, given its role in stress reactivity [96] and prosocial behavior [49]. Contrary to our hypotheses (H4), linear regression analyses did not reveal a significant relationship between CM severity and *OXTR* expression. However, exploratory analysis revealed that lower *OXTR* expression was associated with higher levels of detachment—a maladaptive personality trait characterized by emotional and interpersonal disengagement. Although these associations did not meet our conservative threshold for statistical significance, they may still be of interest. If replicated, this finding may suggest that reduced *OXTR* expression contributes to social withdrawal and affective flattening, which are key features of detachment, potentially representing one pathway through which CM impacts long-term socio-emotional functioning. In addition, we found that CM severity was positively associated with interpersonal sensitivity, social interaction difficulties, and detachment, indicating reduced social functioning.

### Social functioning in CM-exposed individuals

Previous studies have demonstrated that childhood maltreatment (CM) negatively affects social cognition and social functioning [21, 22], as well as the availability of social resources [97]. These social consequences may, in turn, contribute to increased psychological distress by reducing resilience factors [98, 99]. Both studies identified several social functioning impairments in CM-exposed adults, which will be discussed in the following.

A central aspect of social functioning is the ability to trust others [25]. There is growing evidence that CM is associated with decreased interpersonal trust [26, 28], in line with assumptions of cognitive models of PTSD [100, 101]. Study 1 replicated findings of decreased interpersonal trust across all CM subtypes and severity ranges, suggesting reduced trust occurs even in milder CM histories and less-studied subtypes like neglect.

Furthermore, we found that CM was associated not only with lower self-reported trust, but also with increased behavioral distrust, as assessed through the distrust game. We employed both self-report and behavioral measures (Distrust Game) to assess trust comprehensively, reducing recall bias susceptibility [28]. Our findings support economic games’ validity for trust assessment [75], though they may not fully capture real-world interactions [102]. In line with previous research by Hepp et al. [29], we found that CM severity predicted behavioral distrust. This aligns with a group-comparison study by Neil et al. [30], who found that CM-exposed children were less likely to perceive unfamiliar faces as trustworthy. In contrast, Sellnow et al. [103] reported no group differences in a trust game. These discrepancies may reflect methodological differences in how CM is operationalized. In our study, CM was assessed dimensionally, which may have provided more nuanced information than categorical approaches.

Contrary to our hypotheses, interpersonal trust did not significantly moderate the association between CM and romantic relationship attachment. Although prior studies suggest that trauma-exposed individuals may experience lower levels of trust in intimate relationships [104, 105], the role of social cognition in romantic attachment following CM remains unclear. Our findings may reflect the specific operationalization of romantic attachment in our study—namely, attachment-related anxiety and avoidance—which may not directly interact with generalized interpersonal trust. Although we preregistered moderation models for this cross-sectional design, the causality of effects, and potential mediation effects (e.g., CM reducing trust, and therefore interfering with secure attachment) remains an important question that requires separate, preregistered testing with a longitudinal design.

Nevertheless, Study 1 showed that CM severity significantly predicted both attachment-related anxiety and avoidance. Notably, relationship length was not a significant predictor of attachment style. In line with Fraley, Waller and Brennan [33], who argued against the use of categorical attachment classifications (e.g., secure, fearful), we offer an interpretation based on dimensional assessment of attachment. Our findings suggest that individuals with a history of CM are more likely to exhibit insecure romantic attachment patterns. These results are consistent with previous work indicating that CM is associated with insecure attachment across various relational contexts [106]. Moreover, insecure attachment has been shown to mediate the association between CM and reduced relationship quality [32], which frequently involves decreased satisfaction and trust [35, 105]. This is consistent with our finding that attachment insecurity was negatively associated with relationship satisfaction.

There is growing evidence that decreased interpersonal trust contributes to social isolation and loneliness [23, 24]. In line with this, Study 1 found that lower interpersonal trust was associated with increased loneliness, which was also predicted by higher CM severity. Furthermore, loneliness was significantly associated with attachment-related anxiety and avoidance, as well as with lower relationship satisfaction. These findings suggest that individuals with insecure romantic attachment are more likely to experience loneliness than those with secure attachment styles. Prior studies have linked loneliness to reduced perceived social support and increased health impairments [38, 107]. Thus, insecure attachment may contribute to lower resilience through its negative impact on social connectedness.

Findings from Study 2 further support the interpersonal difficulties observed in CM-exposed individuals. Specifically, CM severity was associated with higher levels of interpersonal sensitivity. Although interpersonal sensitivity has been primarily investigated in PTSD populations [108], it has received less attention in individuals with CM histories. Prior research suggests that interpersonal sensitivity may mediate the relationship between trauma exposure and both psychopathology [109] and impaired social functioning [110]. Additionally, CM severity was associated with higher scores in the WHODAS domain social interaction difficulties, consistent with prior work on impaired social functioning among CM-exposed individuals [16], and our findings from Study 1 (e.g., reduced trust).

Taken together, both studies support that CM contributes to impaired social cognition and functioning—reduced trust, insecure attachment, heightened sensitivity, and interaction difficulties. These impairments likely reduce social resource access and increase vulnerability to adverse outcomes.

### Role of OXTR in gene-environment interactions following child maltreatment

Previous literature on the epigenetic effects of CM on OXTR has yielded heterogeneous results and has rarely examined *OXTR* gene expression directly [53, 57]. We found no significant association between CM severity and OXTR, not supporting our hypothesis that CM leads to increased *OXTR* methylation and reduced gene expression. However, given our relatively small sample size, the possibility of a false-negative result cannot be ruled out. Future research should consider other aspects of the oxytocin system beyond *OXTR* expression levels alone. Despite the absence of support for our primary hypothesis, the well-established role of the oxytocin system in promoting prosocial behavior [46, 47] continues to render OXTR a compelling candidate in studies on gene–environment interactions in the context of early adversity.

In an exploratory analysis, we found an association between lower *OXTR* expression and the maladaptive personality trait detachment. This finding is in line with previous research by Simons et al. [111], who reported associations between *OXTR* expression and negative cognitive schemas. However, given methodological differences in study design, sample characteristics, and measurement instruments, direct comparisons should be made with caution. While preliminary, this finding suggests that reduced *OXTR* expression may contribute to emotional disengagement and social withdrawal, which are core features of detachment.

### Limitations and research implications

First, there are limitations related to the recruited samples. Compared to a representative German population [95], our sample—both overall and across individual CM subtypes—showed particularly high levels of maltreatment. Although we aimed to recruit broadly, moderate to severe CM experiences were overrepresented. Participants recruited via the research training group reported higher CM severity than those recruited via social media. Given that extreme CM experiences are relatively rare [112], our findings may not be generalizable. Moreover, our recruitment did not include a non-maltreated comparison group for Study 2. The study was designed to test dimensional, within-group associations between CM severity and OXTR expression rather than categorical group differences. This dimensional approach was chosen to reflect the substantial heterogeneity in type, severity, chronicity, and developmental timing of maltreatment experiences that exists even among CM-exposed individuals. While this strategy is well suited to examining dose–response like relationships, it precludes conclusions about absolute differences in OXTR expression between CM-exposed and non-exposed individuals. Although CM severity showed substantial variability in the present sample, the lack of a non-CM control group means that ceiling or saturation effects cannot be fully ruled out. In addition, restricted variance in CM exposure within an exposed sample can lead to range restriction, which may attenuate observable associations with biological measures. Future studies should therefore integrate dimensional modeling with non-exposed comparison groups or population-based designs to clarify potential non-linearities and threshold effects.

Additionally, participants suffered from various types of psychopathology (see Table S2) that could have affected the results. Consistent with the preregistered dimensional design and to avoid multicollinearity, we did not include psychopathology or diagnoses as covariates in the confirmatory analyses. From a conceptual perspective, psychopathology may, however, function as a mediator or amplifier of CM-related effects, for example by intensifying threat sensitivity, negative social expectations, or withdrawal tendencies. Disentangling these pathways requires longitudinal designs that can explicitly test mediation and reciprocal effects over time. Future studies should therefore integrate repeated assessments of CM-related symptoms and interpersonal functioning to clarify how psychopathology and social-cognitive processes jointly contribute to long-term outcomes following childhood maltreatment.

With regard to the assessment of CM, we used the CTQ as a validated self-report measure. However, retrospective self-reports are susceptible to various cognitive and response-style biases, which can be further amplified by current psychological distress, as shown in previous studies [113, 114]. Moreover, retrospective reports tend to yield stronger associations with psychopathology than prospective reports, possibly due to shared method variance or mood-congruent recall [115, 116].

Another limitation relates to the sociodemographic composition of the sample. Both samples consisted predominantly of young women. Study 1 included mostly cisgender, well-educated individuals without a migration background—a so-called WEIRD sample [117]. These characteristics limit the generalizability of our findings. People of color, LGBTQ+ individuals, and older adults are known to be disproportionately affected by CM and its consequences [118–120] but were clearly underrepresented in our sample.

Second, there are methodological limitations concerning the assessment of interpersonal difficulties. Our studies focused on selected domains and did not include other important aspects, such as perceived social support in PTSD [121], acceptance [122], or closeness to others [123]. According to Pfaltz et al. [124], future research should target difficulties in regulating proximity and distance in close relationships. Additionally, it would be important to assess the quality and size of social networks to examine whether CM leads to social thinning [125].

Another methodological limitation is the reliance on self-report measures, which are prone to various biases (e.g., social desirability, recall bias). Regarding the measurement of romantic relationship attachment, our study did not assess aspects such as communication, intimacy, or partner-specific trust, which are important indicators of relationship quality [35, 105]. Future studies should include these dimensions to better evaluate relationship stability and resilience potential. Although common in CM-exposed individuals [7, 8], personality disorders were not assessed using a structured clinical interview.

The cross-sectional design represents another limitation, as it prevents conclusions about the stability of interpersonal difficulties over time or across situations. Ambulatory assessment (AA) could offer a more dynamic view of interpersonal functioning, as shown in studies with individuals with borderline personality disorder [126]. In line with Pfaltz et al. [124], future research should consider both internal (e.g., mood) and external (e.g., presence of others) contextual factors. In Study 2, exploratory measures of interpersonal sensitivity and social interaction consisted of only a few questionnaire items, limiting their depth. Future research should explore these domains using more comprehensive instruments.

Study 2 examined *OXTR* expression in a relatively small sample (*N* = 92), limiting statistical power to detect small effects. Moreover, *OXTR* expression levels in whole blood were low with relatively high measurement variability, reducing the interpretability of our findings. According to the Human Protein Atlas, OXTR expression in blood is very low to undetectable in whole blood and peripheral blood mononuclear cells, whereas expression is higher in selected non-blood tissues (e.g., breast) and present in specific brain regions [127].

In general, the correlation of peripheral blood and brain gene expression is limited to moderate [128], so blood gene expression may not serve as a proxy for central nervous system processes. In addition, expression of the *OXTR* gene has been associated with immune activation [129], and thus also may be sensitive to variation in leukocyte cell-type composition within blood samples. We did not quantify cell-type proportions or inflammatory markers, which could obscure small effect sizes. Future work should adjust for cell composition (e.g., deconvolution or sorting) and include inflammatory indices. These factors limit the interpretability of associations between peripheral *OXTR* gene expression and brain-related outcomes and should be considered when generalizing the present findings.

Additionally, we did not assess DNA methylation data, which would be essential for investigating gene–environment interactions in relation to gene expression. Combining methylation and expression analyses could maximize the information gained from biosamples. Finally, we were unable to examine the association between *OXTR* expression and social functioning or psychopathology in greater depth. To adequately explore the role of proteins such as OXTR in CM-exposed individuals, future studies should adopt a multimodal approach, combining genotype, epigenetic, and gene expression data. Given the complexity of these interactions and the need for multiple testing corrections, large sample sizes will be essential [53].

## Conclusions

Across two studies, we show that childhood maltreatment is reliably associated with lower interpersonal trust, greater behavioral distrust, and more insecure romantic attachment (higher anxiety and avoidance), with additional links to loneliness and broader social functioning difficulties. These effects emerged across maltreatment subtypes and severity levels and were not explained by demographic covariates. Interpersonal trust did not moderate the association between maltreatment and attachment, suggesting that generalized trust and romantic attachment tap partly distinct aspects of social functioning. In contrast, peripheral OXTR mRNA expression in whole blood was not significantly associated with maltreatment severity. An exploratory association between lower OXTR expression and trait detachment warrants further replication.

Taken together, our findings highlight social-cognitive mechanisms—reduced trust and attachment insecurity—as proximal pathways through which maltreatment may erode relationship quality and social connectedness. Clinically, brief screening of maltreatment history alongside trust, attachment insecurity, and loneliness may inform case formulation and guide trauma-informed interventions that target distrust and attachment-related difficulties. Future work should use larger, more diverse, longitudinal samples and multi-level biological assessments (e.g., genotype, methylation, cell-type–informed expression) to clarify biobehavioral pathways and to test whether improving trust and attachment security translates into better real-world social functioning.

## Supplementary Information

Below is the link to the electronic supplementary material.

Supplementary Material 1

Supplementary Material 2

## Data Availability

The datasets generated during the current study and the analysis code are available at OSF (https://doi.org/10.17605/OSF.IO/4DZWK).

## Abbreviations

BSI: Brief Symptom Inventory
BSI-INS: Interpersonal Sensitivity subscale of the Brief Symptom Inventory
CI: Confidence interval
CM: Childhood maltreatment
CTQ: Childhood Trauma Questionnaire
DG: Distrust Game (behavioral distrust task)
ECR-R: Experiences in Close Relationships – Revised
ECR-Anx: ECR-R Anxiety subscale
ECR-Av: ECR-R Avoidance subscale
GTS: General Trust Scale
OXTR: Oxytocin receptor
PID-5: Personality Inventory for DSM-5
qPCR: Quantitative polymerase chain reaction
SD: Standard deviation
SE: Standard error
UCLA-LS: UCLA Loneliness Scale
WHODAS 2.0: World Health Organization Disability Assessment Schedule 2.0
