# Supplementary material for: Experiences in close relationships, interpersonal trust and OXTR expression in individuals with childhood maltreatment
Source: Borderline Personal Disord Emot Dysregul. 2026 Mar 23;13:9. doi: 10.1186/s40479-026-00340-8 (PMC13063755; doi:10.1186/s40479-026-00340-8)
Supplement: Supplementary file 1 — Supplementary Material 1 [file 40479_2026_340_MOESM1_ESM.docx]

### *Supplemental Materials*

*Table S1.* Demographic details of the final sample of study 1 (n=252).

| *Variable* |  |
| --- | --- |
| *Age at participation* |  |
| Age AM *(SD)* | 31.2 (9.2) |
| Age Range | 18 - 65 |
| *Gender identity* | *N (%)* |
| Female | 233 (92.5%) |
| Male | 15 (5.6%) |
| Non binary | 4 (1.6%) |
| *History of migration* | *N (%)* |
| migration in first or second generation | 54 (21.4%) |
| no migration background | 198 (78.6%) |
| *Highest educational level achieved* | *N (%)* |
| High school diploma | 86 (34.1%) |
| University degree | 60 (23.8%) |
| Other | 106 (42.0%) |
| *Relationship Characteristics* |  |
| Relationship length, AM (SD) | 6.9 (7.7) |
| Relationship length, Range (Years) | 0 - 41 |
| Relationship satisfaction, AM (SD) | 7.4 (2.0) |
| Relationship satisfaction, Range (Years) | 0 - 10 |
| *Sexual Orientation* | *N (%)* |
| Heterosexual | 190 (75.4%) |
| Homosexual | 10 (4.0%) |
| Other | 52 (20.6%) |

*Table S2.* Distributions, Intercorrelations, and Cronbachs alpha of the measured instruments in study 1.

| *Variable* | *M* | *SD* | *Median* | *Min (possible)* | *Max (possible)* | *Cronbach's alpha* | *1* | *2* | *3* | *4* | *5* | *6* |
| --- | --- | --- | --- | --- | --- | --- | --- | --- | --- | --- | --- | --- |
| 1. CTQ | 55.20 | 21.06 | 52.00 | 14.00 (25) | 108 (125) | .95 |  |  |  |  |  |  |
| 2. GTS | 3.05 | 0.77 | 3.00 | 1.00 (1) | 4.83 (5) | .87 | **-.43** |  |  |  |  |  |
| 3. DG | 23.29 | 10.95 | 23.71 | 0.95 (0) | 48.86 (50) | .95 | **.19** | **-.40** |  |  |  |  |
| 4. ECR-Anx | 3.61 | 1.24 | 3.61 | 1.17 (1) | 6.83 (7) | .92 | **.18** | **-.36** | **.27** |  |  |  |
| 5. ECR-Av | 2.96 | 1.29 | 2.72 | 1.00 (1) | 6.22 (7) | .95 | **.37** | **-.45** | **.28** | **.42** |  |  |
| 6. UCLA | 2.41 | 0.85 | 2.40 | 1.00 (12) | 4.85 (5) | .95 | **.37** | **-.61** | **.38** | **.52** | **.66** |  |
| 7. Relationship satisfaction | 7.41 | 2.06 | 7.64 | 1.00 (0) | 10.00 (10) | - | -.15 | **.25** | **-.18** | **-.45** | **-.58** | **-.45** |

*Note*. CTQ = Childhood Trauma Questionnaire, GTS = General Trust Scale, DG = Distrust Game, ECR = Experiences in Close Relationships, ECR-Anx = Anxiety scale, ECR-Av = Avoidance scale; UCLA = University of California Loneliness scale. Correlation coefficients p < .05 in bold font.

Table S3. Linear multiple regression models for the test of Hypothesis 3, moderation effect of Self-reported Trust (GTS)

|  | **Model 5: ECR-Anxiety (GTS x CTQ)** | | **Model 6: ECR-Avoidance (GTS x CTQ)** | |
| --- | --- | --- | --- | --- |
| *Predictors* | *b [95% CI]* | *β [95% CI]* | *b [95% CI]* | *β [95% CI]* |
| Intercept | **3.22 [2.63, 3.82]** |  | **3.07 [2.49, 3.65]** |  |
| CTQ | 0.00 [-0.00, 0.01] | 0.06 [-0.07, 0.19] | 0.01 [0.00, 0.02] | 0.19 [0.06, 0.31] |
| GTS | **0.01 [-0.01, 0.04]** | **0.09 [-0.08, 0.27]** | **0.02 [-0.00, 0.04]** | **0.14 [-0.02, 0.30]** |
| CTQ x GTS | 0.48 [-0.06, 1.01] | 0.10 [-0.01, 0.22] | -0.06 [-0.59, 0.46] | -0.01 [-0.12, 0.10] |
| Age | -0.06 [-0.40, 0.29] | -0.02 [-0.14, 0.10] | -0.07 [-0.41, 0.26] | -0.02 [-0.14, 0.09] |
| Gender | -0.01 [-0.33, 0.30] | -0.01 [-0.13, 0.11] | 0.03 [-0.27, 0.34] | 0.01 [-0.10, 0.13] |
| Sexual orientation | 0.09 [-0.27, 0.45] | 0.03 [-0.09, 0.15] | -0.13 [-0.48, 0.22] | -0.04 [-0.15, 0.07] |
| Educational level | -0.55 [-0.76, -0.34] | -0.34 [-0.47, -0.21] | -0.56 [-0.76, -0.36] | -0.34 [-0.46, -0.21] |
| History of migration | 0.00 [-0.01, 0.01] | 0.04 [-0.07, 0.16] | -0.00 [-0.01, 0.00] | -0.05 [-0.16, 0.06] |
| Relationship length | **-3.91 [-6.60, -1.22]** | **-0.24 [-0.41, -0.07]** | 0.65 [-1.97, 3.27] | 0.04 [-0.12, 0.19] |

*Note.* CTQ = Childhood Trauma Questionnaire, GTS = General Trust Scale, ECR = Experiences in Close Relationships. The covariates were dummy-coded: gender (0=female), sexual orientation (0=heterosexual), educational level (0=high school), history of migration (0=no). Significant results p < .01 in bold.

Table S4. Linear multiple regression models for the test of Hypothesis 3, moderation effect of behavioral distrust (DG)

|  | **Model 7: ECR-Anxiety (DG x CTQ)** | | **Model 8: ECR-Avoidance (DG x CTQ)** | |
| --- | --- | --- | --- | --- |
| *Predictors* | *b [95% CI]* | *β [95% CI]* | *b [95% CI]* | *β [95% CI]* |
| Intercept | **3.32 [2.72, 3.92]** | **—** | **3.18 [2.58, 3.79]** | **—** |
| CTQ | 0.01 [0.00, 0.02] | 0.14 [0.02, 0.26] | **0.02 [0.01, 0.03]** | **0.29 [0.18, 0.41]** |
| DG | **0.02 [-0.01, 0.04]** | **0.12 [-0.05, 0.30]** | **0.02 [0.00, 0.05]** | **0.17 [0.00, 0.34]** |
| CTQ x DG | 0.42 [-0.12, 0.97] | 0.09 [-0.03, 0.21] | -0.10 [-0.65, 0.45] | -0.02 [-0.14, 0.09] |
| Age | -0.18 [-0.53, 0.17] | -0.06 [-0.18, 0.06] | -0.19 [-0.53, 0.16] | -0.06 [-0.18, 0.05] |
| Gender | 0.01 [-0.31, 0.33] | 0.00 [-0.12, 0.13] | 0.05 [-0.27, 0.36] | 0.02 [-0.10, 0.13] |
| Sexual orientation | 0.22 [-0.15, 0.59] | 0.07 [-0.05, 0.19] | -0.01 [-0.38, 0.36] | -0.00 [-0.12, 0.11] |
| Educational level | 0.03 [0.02, 0.04] | 0.27 [0.14, 0.39] | 0.02 [0.01, 0.04] | 0.19 [0.08, 0.31] |
| History of migration | -0.00 [-0.00, 0.00] | -0.06 [-0.18, 0.06] | -0.00 [-0.00, 0.00] | -0.01 [-0.13, 0.10] |
| Relationship length | **-4.43 [-7.16, -1.71]** | **-0.27 [-0.44, -0.10]** | 0.19 [-2.54, 2.92] | 0.01 [-0.15, 0.17] |

*Note.* CTQ = Childhood Trauma Questionnaire, DG = Distrust Game, ECR = Experiences in Close Relationships. The covariates were dummy-coded: gender (0=female), sexual orientation (0=heterosexual), educational level (0=high school), history of migration (0=no). Significant results p < .01 in bold.

Table S5. Linear multiple regression models for the exploratory analysis of potential associations with University of California Loneliness scale (UCLA)

|  | **Model E7: predicting UCLA loneliness** | | | |
| --- | --- | --- | --- | --- |
| *Predictors* | *b [95% CI]* | *β [95% CI]* | *t* | *p* |
| Intercept | **1.29 [0.71, 1.88]** |  | **4.358** | **< .01** |
| CTQ | 0.00 [-0.00, 0.01] | 0.04 [-0.05, 0.13] | 0.831 | 0.41 |
| ECR-Anxiety | **0.17 [0.10, 0.23]** | **0.24 [0.15, 0.34]** | **4.905** | **< .01** |
| ECR-Avoindance | **0.22 [0.15, 0.30]** | **0.34 [0.23, 0.45]** | **6.046** | **< .01** |
| GTS | **-0.34 [-0.45, -0.23]** | **-0.31 [-0.41, -0.21]** | **-6.119** | **< .01** |
| DG | 0.00 [-0.00, 0.01] | 0.06 [-0.03, 0.15] | 1.348 | 0.18 |
| Relationship satisfaction | -0.02 [-0.06, 0.03] | -0.04 [-0.14, 0.07] | -0.732 | 0.46 |
| Age | 0.00 [-0.01, 0.01] | 0.02 [-0.09, 0.14] | 0.404 | 0.69 |
| Gender | -0.05 [-0.30, 0.21] | -0.02 [-0.10, 0.07] | -0.367 | 0.71 |
| Sexual orientation | 0.02 [-0.14, 0.18] | 0.01 [-0.07, 0.09] | 0.266 | 0.79 |
| Educational level | 0.03 [-0.12, 0.18] | 0.02 [-0.06, 0.10] | 0.437 | 0.66 |
| History of migration | -0.03 [-0.20, 0.14] | -0.02 [-0.10, 0.07] | -0.394 | 0.69 |
| Relationship length | 1.13 [-0.16, 2.41] | 0.10 [-0.01, 0.22] | 1.725 | 0.09 |

*Note.* CTQ = Childhood Trauma Questionnaire, ECR = Experiences in Close Relationships, GTS = General Trust Scale, DG = Distrust Game. The covariates were dummy-coded: gender (0=female), sexual orientation (0=heterosexual), educational level (0=high school), history of migration (0=no).

*Table S6.* Demographic details of the final sample of study 2 (n=92).

| *Variable* |  |
| --- | --- |
| *Age at participation* | *N (%)* |
| Age AM *(SD)* | 31.2 (10.89) |
| Age Range | 18 - 60 |
| *Gender identity* | *N (%)* |
| Female | 62 (67.4%) |
| Male | 22 (23.9%) |
| do not want to answer | 8 (8.7%) |
| *History of migration* | *N (%)* |
| migration in first or second generation | 8 (8.7%) |
| no migration background | 76 (82.6%) |
| do not want to answer | 8 (8.7%) |
| *Highest educational level achieved* | *N (%)* |
| Lower secondary school | 5 (5.4%) |
| Intermediate secondary school | 15 (16.3%) |
| Higher education entrance qualification | 63 (68.5%) |
| other | 1 (1.1%) |
| do not want to answer | 8 (8.7%) |
| *Vocational education* | *N (%)* |
| None / semi-skilled | 32 (34.8%) |
| Apprenticeship | 24 (26.1%) |
| Vocational School | 2 (2.2%) |
| University degree | 26 (28.3%) |
| do not want to answer | 8 (8.7%) |
| *Relationship status* | *N (%)* |
| Single | 60 (65.2%) |
| Married | 8 (8.7%) |
| In relationship | 7 (7.6%) |
| divorced | 5 (5.4%) |
| separated | 2 (2.2%) |
| widowed | 2 (2.2%) |
| do not want to answer | 8 (8.7%) |

*Table S7.* Current diagnoses of participants in study 2 (n=92).

| *Diagnosis* | *N (%)* |
| --- | --- |
| *Mood Disorder* |  |
| Major Depression | 57 (62.0%) |
| Biploar I Disorder | 3 (3.3%) |
| *Anxiety Disorder* |  |
| Panic Disorder | 11 (12.0%) |
| Agoraphobia | 7 (7.6%) |
| Social Anxiety Disorder | 19 (20.7%) |
| Generalized anxiety disorder | 4 (4.3%) |
| Obsessive compulsive disorder | 5 (5.4%) |
| Posttraumatic stress disorder | 38 (41.3%) |
| *Other mental disorder* |  |
| Substance use disorder | 17 (18.5%) |
| Eating disorder | 19 (20.7%) |
| Somatic Symptom Disorder | 5 (5.4%) |
| Prämenstrual Dysphoric Disorder | 4 (4.3%) |
| AD(H)D | 15 (16.3%) |
| Brief Psychotic Disorder | 4 (4.3%) |

*Table S8.* Distributions, Intercorrelations, and Cronbachs alpha of the measured instruments in study 2.

| *Variable* | *M* | *SD* | *Median* | *Min (possible)* | *Max (possible)* | *Cronbach's alpha* | *1* | *2* | *3* | *4* |
| --- | --- | --- | --- | --- | --- | --- | --- | --- | --- | --- |
| 1. CT mean | 15.00 | 0.60 | 14.99 | 13.14 | 16.4 | — |  |  |  |  |
| 2. CTQ | 57.64 | 20.81 | 53.5 | 25 | 109 | .94 | –.12 |  |  |  |
| 3. BSI INT | 8.10 | 3.22 | 8.00 | 4 | 17 | .84 | –.18 | **.38** |  |  |
| 4. WHODAS getting along | 7.77 | 3.44 | 7.00 | 4 | 16 | .69 | –.03 | **.34** | **.65** |  |
| 5. PID-5 Detachment | 0.94 | 0.60 | 0.88 | 0 | 2.42 | .87 | –.22 | **.30** | **.56** | **.43** |

*Note*. CT mean = ΔCq (OXTR expression), CTQ = Childhood Trauma Questionnaire, BSI INT = Brief Symptom Inventory, Interpersonal Sensitivity subscale,

WHODAS = WHO Disability Assessment Schedule (Domain 4: getting along with others), PID-5 = Personality Inventory for DSM-5, Correlation coefficients p < .01 in bold font.
