# Supplementary material for: Experiences in close relationships, interpersonal trust and OXTR expression in individuals with childhood maltreatment
Source: Borderline Personal Disord Emot Dysregul. 2026 Mar 23;13:9. doi: 10.1186/s40479-026-00340-8 (PMC13063755; doi:10.1186/s40479-026-00340-8)
Supplement: Supplementary file 2 — Supplementary Material 2 [file 40479_2026_340_MOESM2_ESM.pdf]

Seite 01

E

CO03

Vielen Dank, dass Sie an unserer Studie teilnehmen möchten!

Vorab ein paar wichtige Hinweise:

- Sie müssen, um an dieser Studie teilzunehmen, **mindestens 18 Jahre** alt sein und sich in einer **aktuellen Partnerschaft** befinden.
- Die Bearbeitungszeit beträgt **ca. 25 Minuten.**
- Bitte nutzen Sie die **Pfeiltasten innerhalb des Fragebogenfensters** und nicht die Ihres Browsers.

Im Folgenden erwarten Sie erst einmal weitere Informationen und die Einwilligungserklärung.

Seite 02

Teilnehmerinformation und Information zum Datenschutz

Sehr geehrte*r Studieninteressent*in,

in dieser Studie werden Fragebögen dargeboten, die sich mit Ihrem Verhalten in einer partnerschaftlichen Beziehung beschäftigen, Ihr generelles Vertrauen in andere Menschen erfragen und negative Kindheitserfahrungen erfassen.

Ziel der Studie

Wir interessieren uns dafür, wie sich traumatische Erlebnisse in der Kindheit auf das Vertrauen und die Beziehung zu Ihrem Partner auswirken. Diese Studie wird am Zentralinstitut für Seelische Gesundheit an der Klinik für Psychosomatik und Psychotherapeutische Medizin (Universität Heidelberg) durchgeführt. Die hierbei erhobenen Daten werden ausschließlich dafür genutzt, um das genannte Ziel der Studie zu untersuchen.

Ablauf der Studie

Sollten Sie zu der Teilnahme einwilligen und Ihre Volljährigkeit bestätigen, werden wir Sie zunächst bitten, Fragebögen zu Ihrem Verhalten innerhalb Ihrer Paarbeziehung und zu Ihrem Vertrauen gegenüber anderen Personen zu beantworten. Danach werden Sie ein kurzes Vertrauensspiel mit fiktiven Personen spielen. Zum Schluss bitten wir Sie Fragen zu negativen Kindheitserfahrungen zu beantworten. Das Bearbeiten der Studie dauert insgesamt ungefähr 25 min. Sie können die Beantwortung der Fragebögen jederzeit unterbrechen und zu einem späteren Zeitpunkt fortsetzen, müssen dafür allerdings den Internet-Browser geöffnet halten.

Mögliche Vorteile Ihrer Teilnahme und Freiwilligkeit

Die Teilnahme ist freiwillig. Es entstehen für Sie keinerlei Nachteile, falls Sie nach dieser Aufklärung nicht in die Teilnahme an der Studie einwilligen sollten. Sie können die Teilnahme jederzeit und ohne Angabe von Gründen abbrechen. Durch Ihre Teilnahme entsteht für Sie kein direkter, persönlicher Vorteil. Sie leisten durch Ihre Teilnahme jedoch einen wertvollen Beitrag zu unserer Forschung und tragen zur medizinischen und psychologischen Wissenserweiterung bei.

Sie sind nach Beendigung der Fragebögen für die Teilnahme an einem Gewinnspiel berechtigt, in dem wir unter den 150 Teilnehmer*innen insgesamt 50 Gutscheine im Wert von 20€ verlosen, die online bei über 500 Shops eingelöst werden können (www.wunschgutschein.de). Die Teilnahme an dem Gewinnspiel ist freiwillig. Wenn Sie an dem Gewinnspiel teilnehmen möchten, wird Ihre Email-Adresse getrennt von den restlichen Daten erfasst und nach Beendigung der Verlosung unverzüglich gelöscht.

Nebenwirkungen und Komplikationen

Durch das Ausfüllen der Fragebögen kann es bei manchen Personen vorkommen, dass sie sich vorübergehend aufgewühlt oder stärker belastet fühlen. Es gibt jedoch keine Hinweise auf länger andauernde unerwünschte Effekte.

Sicherheitshinweis: Falls Sie die Studie auf ihrem Smartphone ausführen möchten: Wir weisen Sie darauf hin, dass Sie die Studie nicht bearbeiten dürfen, während Sie aktiv am Straßenverkehr teilnehmen, da dies erhebliche Risiken für Sie und andere Verkehrsteilnehmer birgt.

Aufklärung über den Datenschutz

Alle im Verlauf der Studie erhobenen Daten werden streng vertraulich behandelt und nur anonymisiert erhoben und weiterverarbeitet. Das heißt, es sind keine Rückschlüsse auf Ihre Person möglich. Alle elektronischen Daten werden in anonymisierter und verschlüsselter Form auf einem geschützten Server des Zentralinstituts für seelische Gesundheit in Mannheim gelagert. Die Beachtung des Datenschutzstandards der Europäischen Union ist in vollem Umfange gewährleistet. Die Ergebnisse der o.g. Studie werden ohne jede Bezugsmöglichkeit auf Ihre Person voraussichtlich in medizinisch-psychologischen Fachzeitschriften und auf einem online Archiv (https://osf.io/) veröffentlicht. Ihre anonymisierten Daten werden nach Abschluss der Studie für 10 Jahre in verschlüsselter Form aufbewahrt.

Wir weisen Sie darauf hin, dass aufgrund der Durchführung der Studie in einem Onlineportal keine Garantie für den Schutz personengebundener Daten gemäß DSGVO gegeben werden kann. In der aktuellen Studie werden von uns daher ausdrücklich keine personengebundenen Daten erhoben. Durch die Onlineerhebung ergeben sich dennoch Risiken, dass Daten durch Drittanbieter abgegriffen werden könnten. Durch den Einsatz von Verschlüsselungstechniken und die Speicherung auf einem geschützten Server reduziert sich dieses Risiko, ein Restrisiko bleibt jedoch bestehen.

Die Verantwortlichen für die studienbedingte Erhebung und Auswertung der Daten sind: PD Dr. Inga Niedtfeld (inga.niedtfeld@zi-mannheim.de, Tel.: 0621 1703 4403) Cand. med. Marie Hofmann (marie.hofmann@zi-mannheim.de, Tel.: 0621 1703 4427).

Bei Anliegen zur Datenverarbeitung und zur Einhaltung der datenschutzrechtlichen Anforderungen können Sie sich an folgende Datenschutzbeauftragte der Einrichtung wenden: Dr. Regina Mathes (datenschutzbeauftragter@zi-mannheim.de).

Im Falle einer rechtswidrigen Datenverarbeitung haben Sie das Recht, sich bei der Aufsichtsbehörde zu beschweren: Der Landesbeauftragte für Datenschutz und die Informationsfreiheit Baden-Württemberg Postfach 10 29 32, 70025 Stuttgart Königstraße 10a, 70173 Stuttgart Tel.: 0711/61 55 41 0 Fax: 0711/61 55 41 15 E-Mail: poststelle@lfdi.bwl.de Internet: http://www.baden-wuerttemberg.datenschutz.de

Wenn Sie noch weitere Fragen über den Studienablauf haben oder Ihnen noch etwas unklar ist, wenden Sie sich an die Studienleiterin Frau PD Dr. I. Niedtfeld (Tel.: 0621/1703-4403; inga.niedtfeld@zi-mannheim.de), Zentralinstitut für Seelische Gesundheit, J 5, 68159 Mannheim, Klinik für Psychosomatische und Psychotherapeutische Medizin an der Ruprecht-Karls-Universität Heidelberg.

Die ausführliche Probandeninformation können Sie hier herunterladen und abspeichern:

Probandeninformation herunterladen

CO01

1. Einwilligungserklärung

Der Inhalt und Ablauf dieser Studie sowie mögliche Risiken wurden mir erklärt. Ich habe die Information für Studieninteressierte erhalten, das heißt, ich hatte die Möglichkeit, sie als Datei herunterzuladen und zu speichern oder für mich auszudrucken. Ich bestätige, dass ich die Voraussetzungen für eine Teilnahme erfülle: Ich bin mindestens 18 Jahre alt und befinde mich aktuell in einer partnerschaftlichen Beziehung.

Mir ist bekannt, dass diese Studie in erster Linie der medizinischen und psychologischen Wissenserweiterung dient und keinen persönlichen Vorteil für mich bringt. Ich weiß, dass mir durch Abbruch der Studie oder Nichtbeantwortung einzelner Fragen kein Nachteil entsteht, und dass ich mich bei Nachfragen per E-Mail an die Studienleitung ( ) wenden kann.

Mir ist bekannt, dass bei dieser Studie Daten in anonymisierter Form erhoben werden sollen, das heißt, dass ein Rückschluss auf meine Person nicht möglich ist. Die Weitergabe, Speicherung, und Auswertung dieser studienbezogenen Daten erfolgt nach gesetzlichen Bestimmungen und setzt vor Teilnahme an der Studie folgende freiwillig abgegebene Einwilligungserklärung voraus.
Falls ich mich dazu entscheide, an dem Gewinnspiel teilzunehmen, wird meine Email-Adresse getrennt von den restlichen Daten erfasst und nach Beendigung der Verlosung unverzüglich gelöscht.

Ich willige freiwillig in die Teilnahme an der oben beschriebener Studie ein. Ich willige ein, dass die im Rahmen dieser Studie erhobenen, anonymisierten Daten in der Forschungsabteilung der Klinik für Psychosomatische und Psychotherapeutische Medizin am Zentralinstitut für Seelische Gesundheit Mannheim, Ruprecht-Karls-Universität Heidelberg, gespeichert und durch PD Dr. Inga Niedtfeld und Cand. Med. Marie Hofmann ausgewertet werden. Ich willige ein, dass meine Daten nach Beendigung oder Abbruch der Studie 10 Jahre aufbewahrt werden.

- ○ Ich willige ein
- ○ Ich willige nicht ein

1 aktive(r) Filter

Filter CO01/F1
Wenn eine der folgenden Antwortoption(en) ausgewählt wurde: **2**
Dann nach dem Klick auf "Weiter" den Text **CO02** anzeigen und das Interview beenden

Zunächst würden wir Sie bitten, einige kurze Fragen zu Ihrer Person zu beantworten.

SD01

2. Wie alt sind Sie?

Ich bin [ ] Jahre alt

SD02

3. Wie gut sind Ihre Deutschkenntnisse?

[Bitte auswählen]

SD03

4. Geben Sie im Folgenden bitte an mit welchem Geschlecht Sie sich identifizieren.
Dies kann von Ihrem biologischen Geschlecht abweichen.

- ○ weiblich
- ○ männlich
- ○ divers
- ○ Ich kann und möchte mich keinem Geschlecht zuordnen
- ○ Ich möchte diese Frage nicht beantworten

SD04

5. Wie würden Sie Ihre sexuelle Orientierung beschreiben?

- ○ heterosexuell
- ○ homosexuell
- ○ bisexuell
- ○ asexuell
- ○ pansexuell
- ○ Keines der Genannten trifft auf mich zu.
- ○ Ich möchte diese Frage nicht beantworten.

SD05

6. Geben Sie bitte im Folgenden das Geschlecht Ihres Partners/Ihrer Partnerin an.
Hiermit ist das Geschlecht gemeint, mit dem sich Ihr Partner/Ihre Partnerin identifiziert.

- ○ weiblich
- ○ männlich
- ○ divers
- ○ keine Identifikation
- ○ Ich möchte diese Frage nicht beantworten.

SD06

7. Welchen Bildungsabschluss haben Sie?
Bitte wählen Sie den **höchsten** Bildungsabschluss, den Sie bisher erreicht haben.

- ○ SchülerIn
- ○ Schule beendet ohne Abschluss.
- ○ Hauptschulabschluss/ Volksschulabschluss
- ○ Realschulabschluss/ mittlere Reife
- ○ Abgeschlossene Lehre
- ○ Fachabitur/ Fachhochschulreife
- ○ Abitur/ Hochschulreife
- ○ Bachelorabschluss
- ○ Meisterabschluss
- ○ Masterabschluss/letztes Staatsexamen
- ○ Promotion

SD07

8. Haben Sie einen Migrationshintergrund?

- ☐ Nein
- ☐ Ja, ich bin selbst in einem anderen Land geboren worden [Welches Land?]
- ☐ Ja, meine Mutter und/oder Vater sind in einem anderen Land geboren worden [Welches Land?]
- ☐ Ja, ein oder mehrere Großelternteile sind in einem anderen Land geboren worden [Welches Land?]
- ☐ Ich möchte diese Frage nicht beantworten.

SD08

9. Befinden Sie sich zur Zeit in einer Partnerschaft?

- ○ Ja
- ○ Nein

1 aktive(r) Filter
Filter SD08/F1
Wenn eine der folgenden Antwortoption(en) ausgewählt wurde: **2, -9**
Dann nach dem Klick auf "Weiter" den Text **CO02** anzeigen und das Interview beenden

SD09

10. Seit wie vielen Jahren befinden Sie sich schon in dieser Partnerschaft?

Zeit (in Jahren) [ ]

SD10

11. Wie zufrieden sind Sie im Moment insgesamt mit dieser Partnerschaft?
0 = sehr unzufrieden, 10 = äußerst zufrieden

1	2	3	4	5	6	7	8	9	10
○	○	○	○	○	○	○	○	○	○

EC01

12. Die folgenden Aussagen beziehen sich darauf, wie Sie sich in emotional bedeutsamen Partnerschaften fühlen. Von Interesse ist für uns dabei vor allem, wie Sie Ihre aktuelle Partnerschaft erleben. Bitte prüfen Sie jede Aussage für sich.

	stimme überhaupt nicht zu	stimme nicht zu	stimme eher nicht zu	stimme weder zu noch nicht zu	stimme eher zu	stimme zu	stimme voll und ganz zu
Ich habe Angst, die Liebe meines Partners/meiner Partnerin zu verlieren.							
Ich ziehe es vor, einem Partner/einer Partnerin **nicht** zu zeigen, wie ich in meinem Innersten fühle.							
Ich mache mir oft Sorgen, dass mein Partner/meine Partnerin **nicht** bei mir bleiben will.							
Ich fühle mich wohl damit, meine privaten Gedanken und Gefühle mit meinem Partner/meiner Partnerin zu teilen.							
Ich mache mir oft Sorgen, dass mein Partner/meine Partnerin mich **nicht** wirklich liebt.							
Es fällt mir schwer, mich auf meinen Partner/meine Partnerin zu verlassen.							
Ich mache mir häufig Sorgen um meine Beziehungen.							
Ich fühle mich sehr wohl damit, meinem Partner/meiner Partnerin nahe zu sein.							
Ich wünsche mir oft, dass die Gefühle meines Partner/meiner Partnerin mir gegenüber genauso stark sind, wie meine Gefühle für ihn/sie.							
Ich fühle mich unwohl, mich meinem Partner/meiner Partnerin zu öffnen.							
Ich befürchte, dass ich meinem Partner/meiner Partnerin weniger bedeute, als er/sie mir.							
Ich ziehe es vor, meinem Partner/meiner Partnerin **nicht** nahe zu sein.							
Wenn mein Partner/meine Partnerin **nicht** in meiner Nähe ist, befürchte ich häufig, er/sie könnte sich für jemand anderen interessieren.							
Mir wird unwohl, wenn mein Partner/meine Partnerin mir sehr nahe sein will.							
Wenn ich in einer Partnerschaft Gefühle zeige, habe ich Angst, dass mein Partner/meine Partnerin **nicht** ebenso für mich fühlt.							
Mir fällt es relativ leicht, meinem Partner/meiner Partnerin nahe zu kommen.							
Ich mache mir selten Sorgen darüber, dass mein Partner/meine Partnerin mich verlässt.							
Ich fühle mich wohl dabei, mich auf meinen Partner/meine Partnerin zu verlassen.							
Mein Partner/meine Partnerin bringt mich dazu, an mir selbst zu zweifeln.							
Normalerweise bespreche ich Probleme und Anliegen mit meinem Partner/meiner Partnerin.							
Ich mache mir selten Sorgen, verlassen zu werden.							
Es hilft mir, mich in schwierigen Zeiten an meinen Partner/meine Partnerin zu wenden.							
Ich habe den Eindruck, dass mein Partner/meine Partnerin **nicht** so viel Nähe möchte wie ich.							
Ich erzähle meinem Partner/meiner Partnerin so ziemlich alles.							
Manchmal ändern Partner/Partnerinnen ihre Gefühle für mich ohne ersichtlichen Grund.							
Ich bespreche vieles mit meinem Partner/meiner Partnerin.							
Mein Bedürfnis nach großer Nähe schreckt andere Menschen manchmal ab.							
Ich bin nervös, wenn mein Partner/meine Partnerin mir zu nahe kommt.							
Ich habe Angst, dass sobald ein Partner/eine Partnerin mich näher kennen lernt, er/sie mich **nicht** so mag, wie ich wirklich bin.							
Es fällt mir **nicht** schwer, meinem Partner/meiner Partnerin nahe zu kommen.							
Es macht mich wütend, dass ich von meinem Partner/meiner Partnerin **nicht** die Zuneigung und Unterstützung bekomme, die ich brauche.							
Es fällt mir leicht, mich auf meinen Partner/meine Partnerin zu verlassen.							
Ich befürchte, dass ich **nicht** an andere Leute heranreiche oder im Vergleich mit ihnen schlecht abschneide.							
Es fällt mir leicht, meinem Partner/meiner Partnerin gegenüber liebevoll zu sein.							
Mein Partner/meine Partnerin scheint mich nur dann wahrzunehmen, wenn ich wütend bin.							
Mein Partner/meine Partnerin versteht mich und meine Bedürfnisse wirklich.							

GT01

13. Die folgenden Fragen befassen sich mit Ihrem generellen Vertrauen in andere Menschen. Bitte prüfen Sie jede Aussage für sich.

**Inwiefern stimmen die folgenden Aussagen zu?**	stimme überhauptnicht zu	stimme eher nicht zu	stimme weder zu noch nicht zu	stimme eher zu	stimme voll und ganz zu
Die meisten Menschen sind grundsätzlich ehrlich.	○	○	○	○	○
Die meisten Menschen sind vertrauenswürdig.	○	○	○	○	○
Die meisten Menschen sind grundsätzlich gut und freundlich.	○	○	○	○	○
Die meisten Menschen vertrauen anderen.	○	○	○	○	○
Ich habe Vertrauen in andere Menschen.	○	○	○	○	○
Die meisten Menschen reagieren freundlich, wenn ihnen Vertrauen entgegen gebracht wird.	○	○	○	○	○

UC01

14. Die folgenden Aussagen beziehen Sich auf Ihr persönliches Erleben Ihres sozialen Umfelds. Bitte prüfen Sie jede Aussage für sich.

	stimmt gar nicht	stimmt wenig	stimmt teils-teils	stimmt ziemlich	stimmt völlig
Ich fühle mich wohl mit den Menschen um mich herum.	○	○	○	○	○
Ich habe genug Gesellschaft.	○	○	○	○	○
Ich habe niemanden, an den ich mich wenden kann.	○	○	○	○	○
Ich fühle mich allein.	○	○	○	○	○
Ich habe einen Freundeskreis.	○	○	○	○	○
Ich habe viel gemeinsam mit den Menschen um mich herum.	○	○	○	○	○
Ich fühle mich niemandem nah.	○	○	○	○	○
Die Leute um mich herum haben ganz andere Interessen und Ideen als ich.	○	○	○	○	○
Ich bin ein geselliger Mensch.	○	○	○	○	○
Ich habe Menschen, die mir nahe stehen.	○	○	○	○	○
Ich fühle mich ausgeschlossen.	○	○	○	○	○
Meine Freundschaften sind oberflächlich.	○	○	○	○	○
Niemand kennt mich wirklich.	○	○	○	○	○
Ich fühle mich von den anderen isoliert.	○	○	○	○	○
Ich kann mit anderen zusammen sein, wenn ich das will.	○	○	○	○	○
Es gibt Menschen, die mich wirklich verstehen.	○	○	○	○	○
Ich bin zu viel allein.	○	○	○	○	○
Die anderen Menschen haben es schwer, an mich heranzukommen.	○	○	○	○	○
Ich habe Menschen, mit denen ich sprechen kann.	○	○	○	○	○
Ich habe Menschen, an die ich mich wenden kann.	○	○	○	○	○

Anleitung für das Vertrauensspiel

Bei dieser Aufgabe interessieren wir uns für Ihre Einschätzung in einer hypothetischen Entscheidungssituation. Dabei geht es um Sie und zufällig ausgewählte nicht reale andere Personen, deren Bilder im Folgenden einzeln auf Ihrem Bildschirm erscheinen werden. Zur Vereinfachung nennen wir Ihr Gegenüber nun der "Andere".

Stellen Sie sich bitte vor, dass Sie und der Andere zu Beginn jeweils einen Betrag von 50 € erhalten.

Nur der Andere ist anschließend in der Lage, Ihnen etwas von Ihrem Geld wegzunehmen, ohne dass Sie darauf eine Reaktion zeigen können. Diesen Betrag sollen Sie in Abhängigkeit von der dargestellten Person mehrfach einschätzen. Zum Beispiel:

- Wenn Sie davon ausgehen, dass der Andere Ihnen nichts wegnimmt, erhalten Sie 50 € und der Andere erhält auch 50 €.
- Wenn Sie davon ausgehen, dass der Andere Ihnen 25 € wegnimmt, bleiben für Sie 25 €, der Andere erhält 75 €.
- Wenn Sie davon ausgehen, dass der Andere Ihnen 50 € wegnimmt, bleibt für Sie 0 €, der Andere erhält 100 €.

Treffen Sie bitte bei jeder dargestellten Person erneut eine Einschätzung zwischen 0 und 50 €, indem Sie den Cursor Ihrer Maus über die Linie bewegen. Es werden insgesamt 21 solcher Personen abgebildet, es sind also 21 Einschätzungen erforderlich.

PHP-Code

```
text('dis_instr','align=center');
if (!isset($liste2)) {
  $liste2 = array(
array('R1_30_A_0.jpg'     ,     'TT01'),
array('R1_48_A_3.jpg'     ,     'TT02'),
array('R1_56_A_3.jpg'     ,     'TT03'),
array('R1_62_A_0.jpg'     ,     'TT04'),
array('R1_68_A_3.jpg'     ,     'TT05'),
array('R1_91_A_-3.jpg'    ,     'TT06'),
array('R1_99_A_0.jpg'     ,     'TT07'),
array('R1_116_A_-3.jpg'   ,     'TT08'),
array('R1_00_B_-1.jpg'    ,     'TT09'),
array('R1_02_B_-2.jpg'    ,     'TT10'),
array('R1_06_B_1.jpg'     ,     'TT11'),
array('R1_08_B_-1.jpg'    ,     'TT12'),
array('R1_10_B_2.jpg'     ,     'TT13'),
array('R1_12_B_1.jpg'     ,     'TT14'),
array('R1_15_B_2.jpg'     ,     'TT15'),
array('R1_19_B_-1.jpg'    ,     'TT16'),
array('R1_21_B_-2.jpg'    ,     'TT17'),
array('R1_22_B_-2.jpg'    ,     'TT18'),
array('R1_23_B_2.jpg'     ,     'TT19'),
array('R1_24_B_1.jpg'     ,     'TT20'),
array('R1_28_A_-3.jpg'    ,     'TT21'),
);
  shuffle($liste2);
  registerVariable('liste2');
}
$i = loopPage(21);
registerVariable('i');
html('<div style="text-align: center;">');
html('<img src="'.$liste2[$i][0].'", style = "max-width: 70%">');

question($liste2[$i][1]);
```

text('dis_instr', 'align=center')

Was schätzen Sie: Wie viel Geld wird Ihnen die Person wegnehmen?

PHP-Code

```
$TTfilter = (
valueSum('TT01')+
valueSum('TT02')+
valueSum('TT03')+
valueSum('TT04')+
valueSum('TT05')+
valueSum('TT06')+
valueSum('TT07')+
valueSum('TT08')+
valueSum('TT09')+
valueSum('TT10')+
valueSum('TT11')+
valueSum('TT12')+
valueSum('TT13')+
valueSum('TT14')+
valueSum('TT15')+
valueSum('TT16')+
valueSum('TT17')+
valueSum('TT18')+
valueSum('TT19')+
valueSum('TT20')+
valueSum('TT21')
)/21;
registerVariable("TTfilter");
if ($TTfilter<1.2){
 goToPage("end");
}
```

CT02

Die folgenden Fragen befassen sich mit einigen Erfahrungen während Ihrer Kindheit und Jugend. Auch wenn die Fragen sehr persönlich sind, versuchen Sie bitte, sie so ehrlich wie möglich zu beantworten. Kreuzen Sie dazu für jede Frage an, was am besten beschreibt, wie Sie rückblickend die Situation einschätzen.
Wenn eine Frage für Sie zu belastend ist, müssen Sie diese nicht ausfüllen. Da wir allerdings Ihren Fragebogen bei zu vielen fehlenden Fragen aus der Auswertung nehmen müssen, würden wir Sie bitten möglichst viele Fragen zu beantworten.

**Als ich aufwuchs...**	überhaupt nicht	sehr selten	einige Male	häufig	sehr häufig
...hatte ich nicht genug zu essen.	○	○	○	○	○
...wusste ich, dass sich jemand um mich sorgt und mich beschützt.	○	○	○	○	○
...bezeichneten mich Personen aus meiner Familie als „dumm", „faul" oder „hässlich".	○	○	○	○	○
...waren meine Eltern zu betrunken oder von anderen Drogen „high", um für die Familie zu sorgen.	○	○	○	○	○
...gab es jemanden in der Familie, der mir das Gefühl gab, wichtig und jemand besonderes zu sein.	○	○	○	○	○
...musste ich dreckige Kleidung tragen.	○	○	○	○	○
...hatte ich das Gefühl, geliebt zu werden.	○	○	○	○	○

**Als ich aufwuchs...**	überhaupt nicht	sehr selten	einige Male	häufig	sehr häufig
...glaubte ich, dass meine Eltern wünschten, ich wäre nie geboren.	○	○	○	○	○
...wurde ich von jemandem aus meiner Familie so stark geschlagen, dass ich zum Arzt oder ins Krankenhaus musste.	○	○	○	○	○
...gab es nichts, was ich an meiner Familie ändern wollte.	○	○	○	○	○
...schlugen mich Personen aus meiner Familie so stark, dass ich blaue Flecken oder Schrammen davontrug.	○	○	○	○	○
...wurde ich mit einem Gürtel, einem Stock, einem Riemen oder mit einem harten Gegenstand bestraft.	○	○	○	○	○
...gaben meine Familienangehörigen aufeinander acht.	○	○	○	○	○
...sagten Personen aus meiner Familie verletzende oder beleidigende Dinge zu mir.	○	○	○	○	○

**Als ich aufwuchs...**	überhaupt nicht	sehr selten	einige Male	häufig	sehr häufig
Ich glaube, ich bin körperlich misshandelt worden, als ich aufwuchs.	○	○	○	○	○
...hatte ich eine perfekte Kindheit.	○	○	○	○	○
...wurde ich so stark geschlagen oder verprügelt, dass es jemandem (z.B. Lehrer, Nachbar oder Arzt) auffiel.	○	○	○	○	○
...hatte ich das Gefühl, es hasste mich jemand in meiner Familie.	○	○	○	○	○
...fühlten sich meine Familienangehörigen einander nah.	○	○	○	○	○
...versuchte jemand, mich sexuell zu berühren oder mich dazu zu bringen, sie oder ihn sexuell zu berühren.	○	○	○	○	○
...drohte mir jemand, mir weh zu tun oder Lügen über mich zu erzählen, wenn ich keine sexuellen Handlungen mit ihm oder ihr ausführen würde.	○	○	○	○	○

**Als ich aufwuchs...**	überhaupt nicht	sehr selten	einige Male	häufig	sehr häufig
...hatte ich die beste Familie der Welt.	○	○	○	○	○
...versuchte jemand, mich dazu zu bringen, sexuelle Dinge zu tun oder bei sexuellen Dingen zuzusehen.	○	○	○	○	○
...belästigte mich jemand sexuell.	○	○	○	○	○
Ich glaube ich bin emotional (gefühlsmäßig) missbraucht worden, als ich aufwuchs.	○	○	○	○	○
...gab es jemanden, der mich zum Arzt brachte, wenn ich es brauchte.	○	○	○	○	○
Ich glaube ich bin sexuell missbraucht worden, als ich aufwuchs.	○	○	○	○	○
...war meine Familie mir eine Quelle der Unterstützung.	○	○	○	○	○

Vielen Dank für Ihre Teilnahme!

Wir möchten uns ganz herzlich für Ihre Mithilfe bedanken.

Sie können das Browser-Fenster nun schließen.
